# Serial Lung Magnetic Resonance Imaging to Monitor Disease Progression in a Child With a Diffuse Alveolar Hemorrhage Syndrome

**DOI:** 10.14740/jocmr1962w

**Published:** 2015-02-09

**Authors:** Mohammed Kaleel, Craig Schramm, Melanie Pascal, Michael O’Louglin, Melanie Sue Collins

**Affiliations:** a Department of Radiology, Hartford Hospital, Hartford, CT, USA; b Department of Pediatric Pulmonary Medicine, Connecticut Children’s Medical Center, Hartford, CT, USA; c Department of Pediatrics, University of Connecticut Health Center, Farmington, CT, USA; d Tulane University School of Medicine, New Orleans, Louisiana, USA

**Keywords:** Interstitial lung disease, Children, MRI, CT scan, Monitoring, Diffuse alveolar hemorrhage

## Abstract

Serial lung magnetic resonance imaging (MRI) was performed in a child with diffuse alveolar hemorrhage (DAH). To minimize radiation exposure with conventional serial chest computerized tomography (CT), serial MRIs of the lungs were used. This effectively monitored her disease process as well as detected acute hemorrhage after 5 years remission.

## Introduction

Idiopathic pulmonary capillaritis (PC) is a rare disease presenting with symptoms of diffuse alveolar hemorrhage (DAH) and diagnosed by lung biopsy [1, 2]. Careful monitoring is required for patients with PC. Pulmonary function testing (PFT), including DLCO, is recommended [3, 4] but is limited by the age and technique of the child. Computerized tomography (CT) is recommended for diagnosis and monitoring for recurrence of DAH [3]. However, there are concerns regarding radiation exposure in pediatric patients with medical imaging [5]. Because of the nontrivial radiation exposure associated with CT, patients requiring ongoing monitoring with CT scans, such as DAH syndromes, pose a clinical problem. Magnetic resonance imaging (MRI) may play an essential role given its ability to pick up areas of hemorrhage within the lungs with similar sensitivity and specificity to CT, allowing pediatric patients to avoid unnecessary radiation [6]. Based on that case report [6] and the desire to minimize radiation exposure, we opted to use serial MRIs to follow our patient with PC.

## Case Report

**Figure 1:**
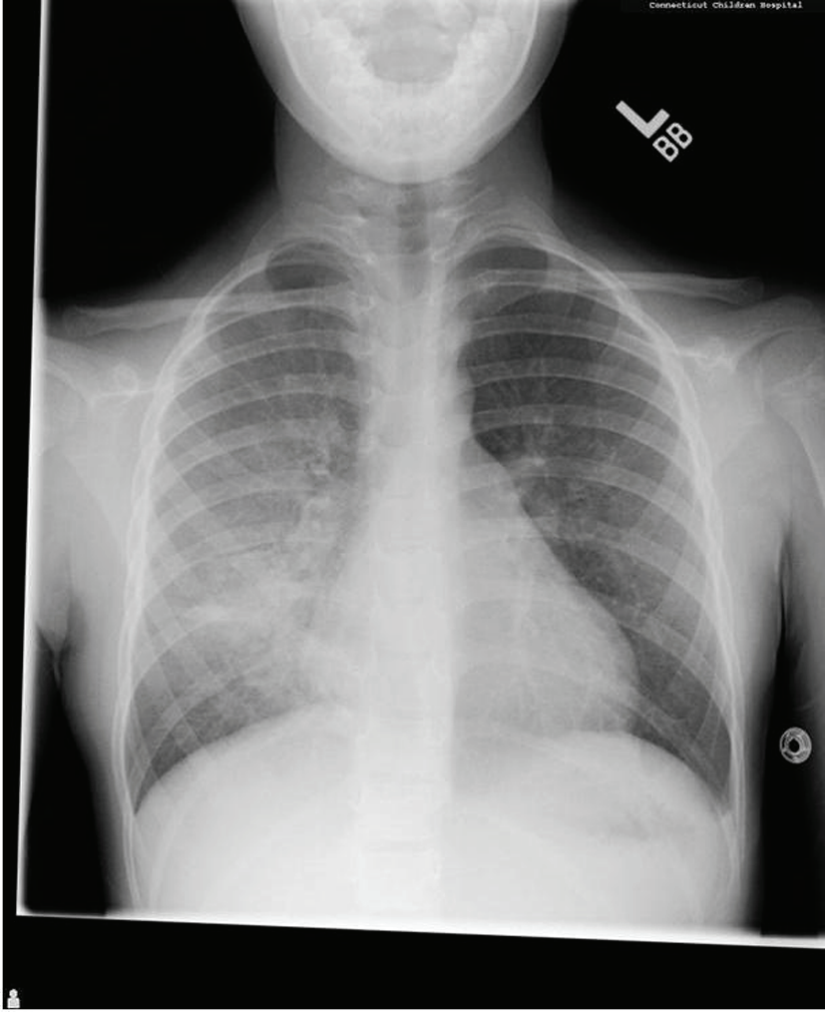
Initial chest X-ray demonstrated a diffuse airspace filling process throughout the right lung and a small airspace opacity in the left upper lobe.

**Figure 2:**
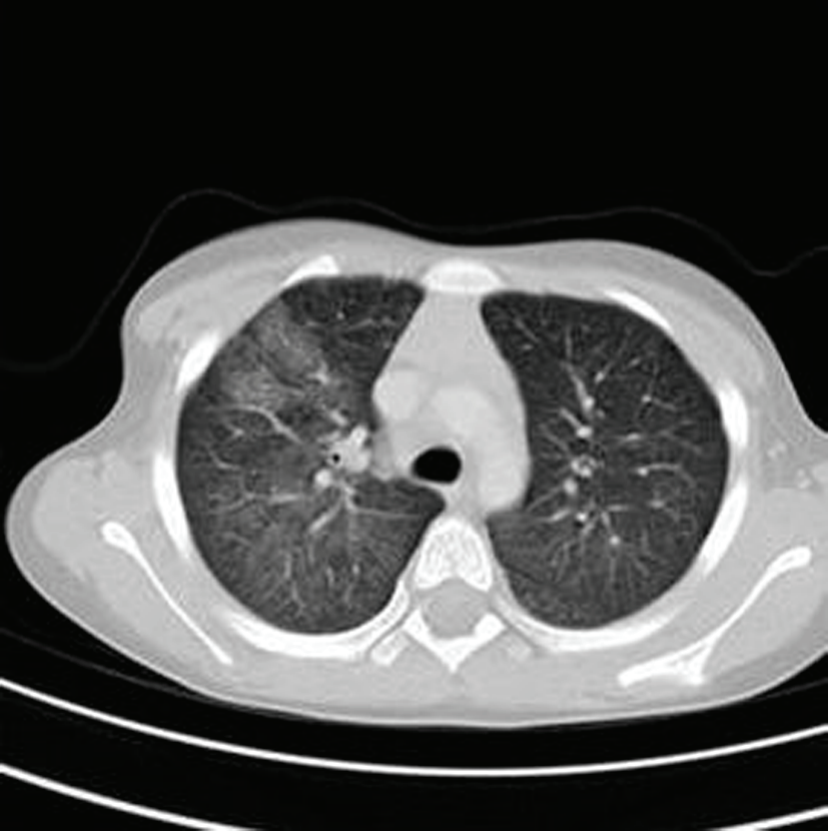
Initial chest CT demonstrated the extent of the airspace filling process. Ground glass opacities were seen in all lobes of the right lung with ground glass opacity identified in the left upper and left lower lobes to a lesser extent. In addition, there is dense right middle lobe consolidation.

A 5-year-old female presented to our hospital in 2007 with a 3 - 4 month history of hemoptysis. Laboratory analysis demonstrated severe anemia, and a chest radiograph showed diffuse airspace opacities in the right lung with a small airspace opacity in the left upper lobe (Fig. 1). CT scan revealed ground glass opacity in all lobes of the right lung with ground glass opacity identified in the left upper and left lower lobes to a lesser extent, as well as dense right middle lobe consolidation (Fig. 2). Subsequent wedge biopsy of the right middle lobe demonstrated pulmonary hemorrhage with changes suggestive of pulmonary capillaritis. Testing for cardiac, rheumatologic, and infectious disease was negative. After cessation of hemoptysis and stabilization of her hemoglobin, she was sent home on prednisolone (1 mg/kg) and hydroxychloroquine. Within 1 month, hemoptysis recurred, and imaging was pursued to evaluate for active bleeding. In order to avoid more radiation exposure with subsequent CT, an MRI was performed to evaluate for pulmonary hemorrhage. MRI revealed increasing opacities in the left upper lobe compared to the prior CT, suggesting increased hemorrhage. Due to young age and ability, she was not able to perform comprehensive PFT at the time of initial diagnosis.

**Figure 3:**
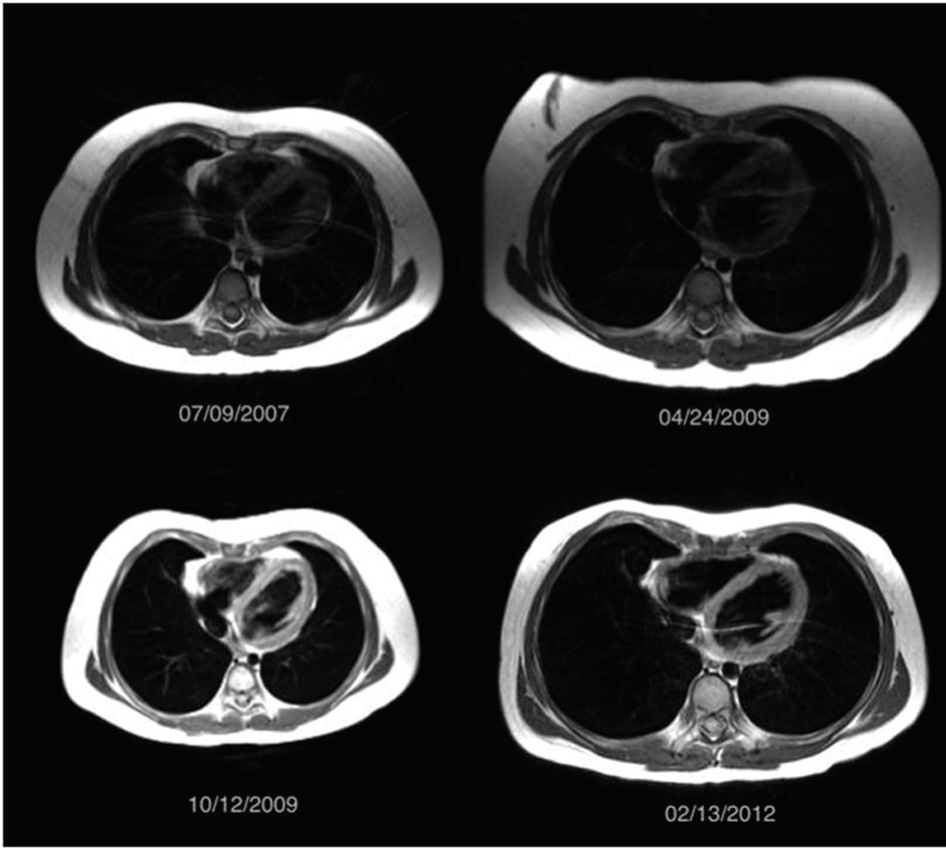
Multiple surveillance MRI scans obtained between July 2007 and February 2012 demonstrated clear lungs without evidence of an abnormal airspace filling process. The patient was asymptomatic during this time period.

Intravenous methylprednisolone (30 mg/kg daily × 3 days) and intravenous immunoglobulin (IVIG) (2 g/kg) were initiated, and then continued weekly and monthly respectively. As MRI demonstrated pulmonary hemorrhage before serologic changes, serial MRIs were performed to monitor disease progression and recurrence, especially as medications were weaned. Over a 5-year period, medications were weaned to monthly IVIG (1 g/kg) and prednisolone (5 mg every other day). With increasing age, her ability to perform more complex PFT improved, and demonstrated no evidence of restriction, obstruction, or diffusion limitation. Serologic markers remained negative, save for a modest increase in ANA (1:160 - 1:320). From July 2007 to February 2012, serial MRI images demonstrated stable disease. Figure 3 shows representative images during that time period.

**Figure 4:**
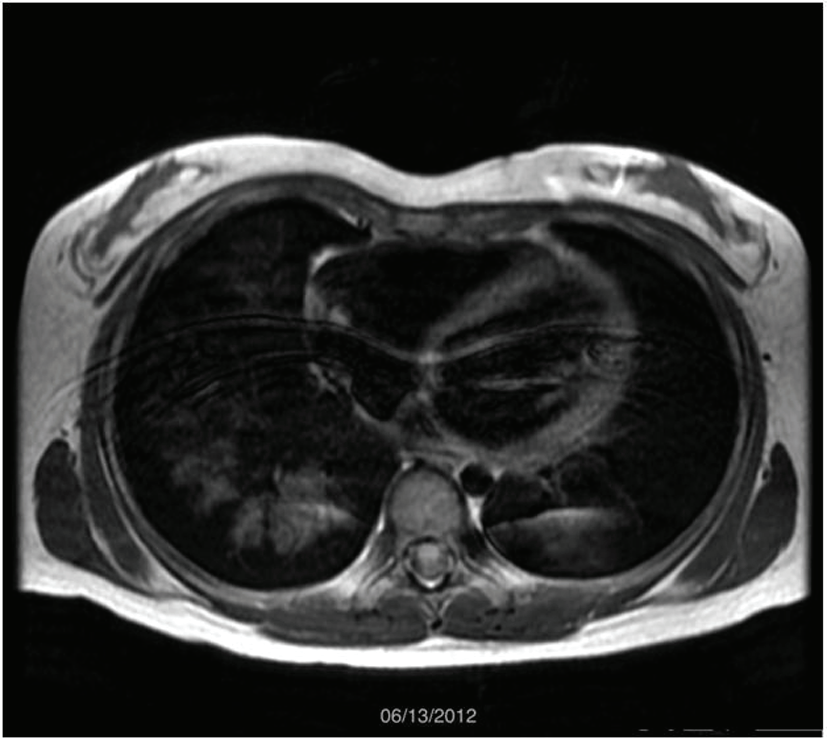
MRI scan obtained on June 2012 demonstrated diffuse airspace opacities in the right lower lobe and left lower lobe. Clinically, the patient was experiencing hemoptysis and found to be in an exacerbation of her condition.

**Figure 5:**
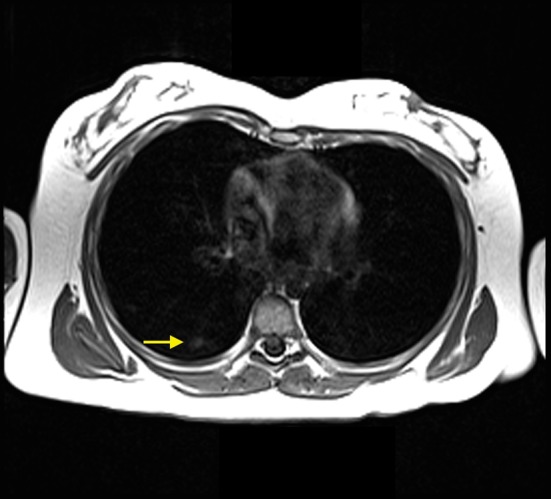
MRI scan obtained November 2012. Axial T1 image showed multiple small peripheral airspace opacities in the right lower lobe (yellow arrow), which was compatible with alveolar hemorrhage given the patient’s history and clinical presentation.

After 5 years of remission, she had recurrent hemoptysis, bilateral radiographic infiltrates, and decreasing hemoglobin. PFTs revealed an increase in DLCO consistent with an acute pulmonary hemorrhage. Repeat MRI revealed new patchy airspace opacities involving the majority of the right lung and the left lower lobe (Fig. 4). As recommendations had changed [4], 1 g weekly intravenous methylprednisolone therapy was added to the monthly IVIG. Five months later, her hemoptysis recurred with similar laboratory and PFT changes. MRI revealed multiple peripheral small airspace opacities in the right lung (Fig. 5). IVIG was increased to 2 g/kg monthly, and azathioprine was started (2 mg/kg/day). She is stable on that regimen with normal PFTs and unchanged serologic markers.

## Discussion

The patient demonstrates a typical case of idiopathic PC. Decreasing the morbidity (i.e. risk of malignancies) associated with long-term radiation exposure from multiple monitoring CT scans is a priority, especially, because immunosuppressive therapy for PC has significant short-term and long-term morbidity. The initial MRI demonstrated a new pattern of airspace disease compatible with increased areas of hemorrhage. With the definitive diagnosis obtained from biopsy and the ability to visualize the correlating airspace filling process on MRI, it became possible to follow the hemorrhaging via serial MRI. Although MRI is not typically used for the lungs due to the inability to visualize the normally air filled alveoli on MRI, a diseased lung, such as occurs in a patient with alveolar hemorrhage, can be seen on MRI as a nonspecific airspace filling process, similar to findings on a CT scan. With knowledge of the diagnosis, monitoring the disease with MRI became possible. Following the initial MRI, the patient had subsequent MRIs every 4 - 6 months to look for areas of recurrent airspace hemorrhage. During the 5 years of remission, these images showed a persistent scar in the right middle lobe with little to no evidence of airspace disease. However, the MRI obtained at the time of the recurrent hemoptysis after the 5-year remission demonstrated extensive patchy opacities in the right lung and left lower lobe similar in appearance to the initial CT scan obtained 5 years prior.

To our knowledge, MRIs have not been used to follow children with recurrent pulmonary hemorrhage. In this 5-year period, the patient had 12 MRIs. Had CT scans been used, the amount of radiation received would have been significant. Although there is limited research evaluating risk of malignancy associated with medical imaging, a retrospective cohort study found a clear dose-response relationship [7]. That study reported cumulative doses of 50 - 60 milligrays (mGy) to the head resulted in a three-fold increase in the risk of brain tumors. It also found the same cumulative dose to the bone marrow was associated with a three-fold increase in the risk of leukemia. A pediatric chest CT delivers approximately 3 - 4 mGy of radiation [8]. Had our patient received CT scans instead of MRIs, she would have had a cumulative dose of approximately 36 - 48 mGy.

Due to the variable course of PC, patients must be closely monitored for recurrent bleeding. Furthermore, gradually weaning therapy over time due to deleterious side effects is imperative. We used MRI to monitor the patient’s disease status during the weaning period with success. In our case, serial MRI of the chest provided safe, adequate monitoring and may be considered for monitoring other children with DAH syndromes.
